# Identification and Drought-Responsive Expression Analysis of the *ZmSPS* Gene Family in Maize and Preliminary Investigation of the *ZmSPS3* Regulatory Network

**DOI:** 10.3390/plants15060885

**Published:** 2026-03-12

**Authors:** Minghao Sun, Wei Zhao, Shuai Hou, Haoxin Meng, Luyao Wang, Erna Wu, Enhao Zhou, Yuyang Duan, Yue Wang, Quan Cai, Baitao Guo, Tao Yu, Jianguo Zhang

**Affiliations:** 1 Heilongjiang Academy of Agricultural Sciences, Harbin 150086, China; 2 Postdoctoral Innovation Practice Base, Maize Research Institute, Heilongjiang Academy of Agricultural Sciences, Harbin 150028, China; 3 Key Laboratory of Biology and Genetics Improvement of Maize in Northern Northeast Region, Ministry of Agriculture and Rural Affairs, Harbin 150086, China; 4 Heilongjiang Province Agricultural Techology Extension Station, Harbin 150036, China

**Keywords:** drought stress, maize, *ZmSPS* gene family, protein kinase

## Abstract

Sucrose phosphate synthase (SPS) is a key rate-limiting enzyme that regulates carbon partitioning and stress tolerance in plants. In this study, we systematically characterized the *SPS* gene family in maize (*Zea mays* L.) and identified key members and their interaction networks involved in drought responses. A total of seven *ZmSPS* genes were identified through genome-wide bioinformatics analyses. Motif composition, gene structure, phylogenetic relationships, and synteny analyses indicated that the *ZmSPS* gene family is highly conserved among monocot species. Promoter analysis revealed that the upstream regions of *ZmSPS* genes are enriched with multiple stress responsive cis-acting elements. Drought stress treatments combined with quantitative real-time PCR (RT-qPCR) analyses showed that the expression of *ZmSPS3* was significantly upregulated with increasing stress duration. Furthermore, yeast two-hybrid assays demonstrated that ZmSPS3 physically interacts with protein kinases and F-box proteins. These interactions suggest a potential involvement of *ZmSPS3* in post-translational modification and protein stability regulation during osmotic stress. As a potential candidate gene responsive to drought, *ZmSPS3* provides a preliminary basis for understanding the complex drought-response networks in maize.

## 1. Introduction

Maize (*Zea mays* L.) is one of the most widely produced food crops worldwide, ranking first in both planting area and total production, and it also serves as an important source of animal feed and industrial raw materials [1]. As a major food resource for humans, maize provides essential carbohydrates and proteins and is extensively used in the production of biofuels, starch, and other industrial products [2,3]. The growth, development, and yield of maize are highly sensitive to environmental factors such as water availability, light conditions, and nutrient supply. Among these factors, drought stress is a major abiotic constraint that severely limits maize growth and productivity [4,5,6]. Drought stress suppresses photosynthesis and metabolic activities and disrupts cellular osmotic balance, thereby directly affecting the synthesis and allocation of photosynthetic assimilates and ultimately reducing crop yield [7,8]. During this process, sucrose, as the primary product of photosynthesis, functions not only as an energy source but also as a key molecule involved in osmotic regulation and signal transduction. Sucrose therefore plays a crucial role in maintaining cellular homeostasis and enhancing drought tolerance in maize [9].

In higher plants, sucrose is primarily produced through photosynthesis. The dynamic balance between its synthesis in source organs, transport via the phloem to sink organs, and utilization in sink tissues collectively determines plant growth, development, and final crop yield [10]. Analyzing crop yield improvement from the perspective of source-sink regulation has become a mainstream approach in recent years [11]. In addition to serving as a primary carbon and energy source, sucrose also functions as a signaling molecule, widely participating in the regulation of plant growth, development, and various physiological processes, thereby profoundly affecting crop yield [12,13]. The source-to-sink transport of photosynthetic products in plants involves several key steps: (1) CO_2_ fixation in chloroplasts; (2) transport of carbon assimilation products to the cytoplasm; (3) sucrose synthesis; and (4) sucrose transport to sink tissues and its utilization therein [14,15].

During this process, intermediates generated from CO_2_ fixation via carbon assimilation pathways are ultimately converted into sucrose through the coordinated actions of sucrose-phosphate synthase (SPS) and sucrose-6-phosphate phosphatase (SPP) [16]. Specifically, SPS catalyzes the transfer of a glucose moiety from uridine-5′-diphosphate glucose (UDPG) to fructose-6-phosphate (F-6-P), producing sucrose-6-phosphate (Suc-6-P). Subsequently, Suc-6-P is dephosphorylated by SPP to form sucrose, which is transported from source organs to sink tissues via the phloem to meet the carbon and energy demands required for plant growth, development, and stress adaptation [17,18].

Sucrose-phosphate synthase (SPS) is a soluble enzyme localized in the cytoplasm, with a molecular weight of approximately 120 kDa and an optimal reaction pH of around 7.0. It plays a central catalytic role in plant sucrose biosynthesis [19,20]. Based on phylogenetic analysis, the plant *SPS* gene family can be classified into four subfamilies: A, B, C, and D. Notably, the D subfamily has so far been identified only in monocotyledonous grasses, indicating a certain degree of species specificity [20,21]. Protein structure analysis shows that SPS proteins typically consist of multiple highly conserved functional domains, including the sucrose synthase domain (sucrose-synth), the glycosyltransferase domain (glycos-transf-1), and the sucrose-6-phosphate phosphatase domain (S6PP) [22,23]. These conserved domains collectively form the structural basis of *SPS* and are critical for its catalytic activity and physiological functions.

Previous studies have demonstrated that *SPS* plays a crucial role in plant growth, development, and responses to abiotic stress. In *Arabidopsis thaliana* (Arabidopsis), genetic functional analyses of the *AtSPS* gene family revealed that the *atspsa1/atspsc* double mutant and the *atspsa1/atspsa2/atspsc* triple mutant exhibited pronounced dwarf phenotypes during both vegetative and reproductive stages, with severely impaired development of shoots, floral organs, and siliques. Further studies showed that the *atspsa1/atspsb/atspsc* triple mutant and the quadruple mutant caused defective seed germination [24]. Moreover, overexpression of *AtSPSA1* in Arabidopsis significantly increased sucrose content in stems and was accompanied by enhanced stem height, fiber length, and overall plant height [25].

In maize, comparative studies of varieties with different growth rates have shown a significant positive correlation between *SPS* activity and plant growth rate. This suggests that the capacity to convert photosynthetically produced carbohydrates into sucrose, regulated by SPS, may be an important limiting factor determining early growth in maize [21]. Moreover, under high-temperature stress, inhibition of SPS and other enzymes involved in sucrose metabolism and starch synthesis markedly reduces starch accumulation and kernel weight, further highlighting the critical role of *SPS* in maize grain filling and yield formation. In *Manihot esculenta*, *MeSPS* genes are highly expressed in source tissues, such as leaves and stems, and their expression levels are significantly positively correlated with root starch content. High expression of *MeSPS* accelerates starch accumulation in cassava tuberous roots [26]. In *Solanum lycopersicum*, overexpression of *SlSPS* significantly increases SPS activity and sucrose content, thereby promoting plant growth and enhancing tolerance to high-temperature stress. Conversely, knockout of *SlSPS* suppresses growth, reduces sucrose metabolism, and increases sensitivity to heat stress [27]. Similarly, in *Nicotiana tabacum*, *NtSPS5* and *NtSPS6* have been identified as positive regulators in drought stress responses, contributing to enhanced drought tolerance [28]. Collectively, studies across different species consistently indicate that *SPS* plays a conserved and crucial role in plant growth and development, yield formation, and abiotic stress adaptation by regulating sucrose synthesis and the allocation of photosynthetic assimilates.

To date, studies on maize *SPS* have primarily focused on the identification of gene numbers, and systematic investigations of their functions and regulatory mechanisms remain lacking [20,29]. In this study, we systematically identified members of the maize *SPS* gene family using bioinformatics approaches and conducted comprehensive analyses of their physicochemical properties, promoter elements, evolutionary relationships, and synteny. Additionally, potential functions of *ZmSPS* genes were preliminarily explored through miRNA target prediction and Gene Ontology (GO) annotation. Combined with transcriptome data under drought stress and validation by RT-qPCR, we further characterized the transcriptional response patterns of *ZmSPS* genes. Moreover, yeast two-hybrid library screening and pairwise interaction assays were employed to investigate the potential regulatory pathways of *ZmSPS* under drought stress. This study provides important insights into the role of sucrose metabolism in maize drought tolerance and has significant implications for improving water-use efficiency and enhancing crop yield.

## 2. Results

### 2.1. Identification and Physicochemical Properties of the Maize ZmSPS Gene Family

The *ZmSPS* gene family members in maize were identified using two complementary approaches. First, previously reported studies were combined with the SPS HMM profiles (PF00534, PF00862, PF05116) to search the maize whole genome and obtain candidate genes. Second, known *SPS* members in rice were used to query maize protein sequences, yielding additional candidate genes. The candidate genes obtained from the two methods were then merged. The merged candidates were manually screened for the presence of *SPS* core domains using multiple databases, including CDD, Phmmer, Pfam, and SMART. Ultimately, seven *ZmSPS* genes were confirmed and named *ZmSPS1-7* according to their physical positions on the chromosomes. Analysis of their encoded proteins revealed that the CDS lengths ranged from 2532 bp (*ZmSPS7*) to 3237 bp (*ZmSPS1*), corresponding to protein lengths of 844–1079 amino acids (Table 1). The predicted molecular weights ranged from 94.50 to 119.42 kDa, with minor differences among family members, indicating high structural conservation. The theoretical isoelectric points (pI) of ZmSPS proteins mainly ranged from 6.04 to 7.38, with ZmSPS4 (pI = 7.38) and ZmSPS5 (pI = 7.03) being neutral or slightly basic, while the other 5 members were slightly acidic. Hydropathicity analysis showed that all ZmSPS proteins had negative GRAVY values, ranging from −0.20 to −0.42, suggesting that the family members are overall hydrophilic. Subcellular localization prediction indicated that all seven ZmSPS proteins are cytoplasmic, consistent with the physiological role of sucrose synthesis occurring primarily in the cytoplasm, further supporting the critical role of *ZmSPS* genes in maize carbon metabolism.

### 2.2. Gene Structure and Conserved Motif Analysis of the ZmSPS Gene Family

**Figure 1:**
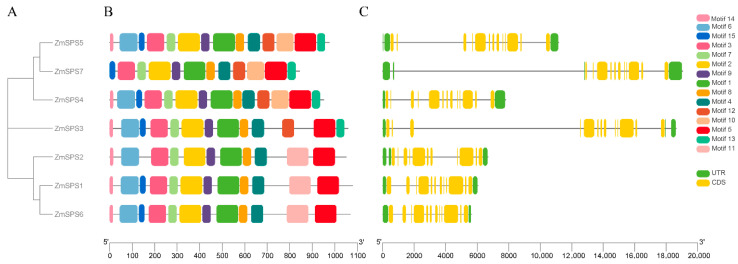
Conserved motifs and gene structure of the maize *ZmSPS* family. (**A**). Phylogenetic tree of ZmSPS proteins, (**B**). Schematic representation of conserved motifs. Different colors indicate distinct motifs. (**C**). Gene structure analysis. Green boxes represent coding sequences (CDS), and yellow boxes represent untranslated regions (UTRs).

To further investigate the evolutionary relationships and structural features of the maize *SPS* gene family, a phylogenetic tree of ZmSPS proteins was constructed, and conserved motifs and gene structures were comprehensively analyzed (Figure 1). Phylogenetic analysis revealed that the seven *ZmSPS* members could be clearly divided into three major evolutionary clades (Figure 1A). Clade I included *ZmSPS5*, *ZmSPS7*, and *ZmSPS4*; Clade II contained only *ZmSPS3*, showing a relatively independent evolutionary position; and Clade III comprised *ZmSPS2*, *ZmSPS1*, and *ZmSPS6*. Members within the same clade were more closely related, suggesting that they may have similar or related biological functions. Conserved motif analysis indicated that *ZmSPS* family members generally possess highly conserved structural features (Figure 1B). All ZmSPS proteins contained most of the same motifs (motifs 1, 2, 3, 4, 5, 7, 8, 9), and the order of these motifs was highly consistent across different members, reflecting strong structural stability during evolution. However, certain motif differences were observed among the evolutionary clades, showing branch-specific patterns. For example, members of Clade III (ZmSPS1, ZmSPS2, and ZmSPS6) uniquely contained motif 11 at the C-terminal, which was absent in Clades I and II. Additionally, ZmSPS7 lacked motif 14 at the N-terminal, and the position of motif 15 differed from other members, suggesting potential functional divergence or specialization.

Gene structure analysis showed that all *ZmSPS* genes contained multiple exons and introns (Figure 1C, Table S2), but the numbers varied. *ZmSPS1*, *ZmSPS2*, and *ZmSPS6* had 12 exons and 11 introns; *ZmSPS4* and *ZmSPS7* had 13 exons and 12 introns, while *ZmSPS3* and *ZmSPS5* contained 14 exons and 13 introns. Overall, genes within the same clade displayed similar structures, reflecting evolutionary conservation of the family, while structural variations among different clades may contribute to functional divergence.

### 2.3. Cis-Acting Element Analysis of ZmSPS Promoters

**Figure 2:**
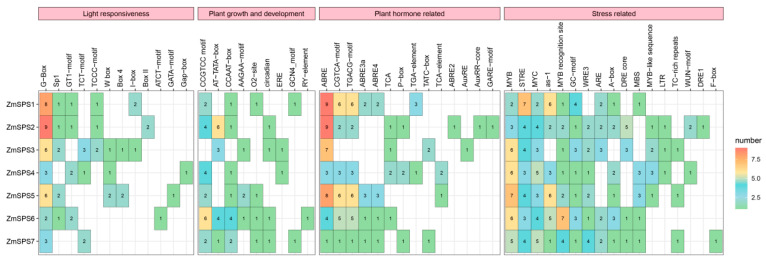
Distribution of *cis*-acting elements in the promoters of maize *ZmSPS* genes. Numbers in the figure represent the copy number of each *cis*-acting element. Red indicates a higher number, while green indicates a lower number. Pink rectangles denote the classification of promoters.

To explore the transcriptional regulatory mechanisms of the maize *ZmSPS* gene family, the 2000 bp upstream sequences of all seven *ZmSPS* genes were extracted and analyzed for *cis*-acting elements (Figure 2). The results showed that the promoter regions were enriched with elements related to light responsiveness, hormone regulation, stress response, and growth and development. Notably, light-responsive elements (G-Box), hormone-responsive elements (ABRE), and stress-related elements (MYB, STRE, MYC, GC-motif) were present in all members. Among them, ABRE elements were most abundant in *ZmSPS1*, *ZmSPS2*, *ZmSPS3*, and *ZmSPS5*, with 9, 9, 7, and 8 copies, respectively, suggesting that these four genes may play central roles in ABA-mediated abiotic stress responses. The light-responsive core element G-Box was highly represented in *ZmSPS1* and *ZmSPS2* promoters, with 8 and 9 copies, respectively, consistent with the role of *SPS* in photosynthate metabolism. *ZmSPS1* also contained a relatively high number of STRE elements. Overall, the high enrichment of ABRE and G-Box elements in the *ZmSPS* promoters indicates that transcriptional regulation of this gene family is primarily driven by light and ABA signaling.

### 2.4. Chromosomal Distribution and Synteny Analysis of ZmSPS Genes

**Figure 3:**
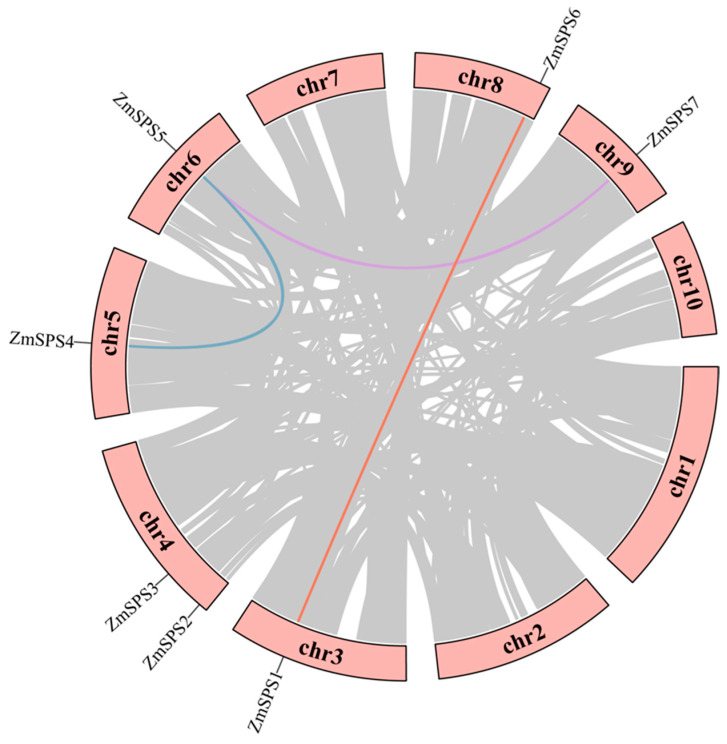
Chromosomal localization and syntenic relationships of the maize *ZmSPS* gene family. The outer circle represents the 10 maize chromosomes (chr1–chr10), with *ZmSPS1*-*ZmSPS7* indicating the positions of family members. Gray lines represent whole-genome or segmental duplication events, while colored lines highlight syntenic *ZmSPS* gene pairs.

To elucidate the physical locations and expansion mechanisms of the maize *ZmSPS* gene family, chromosomal mapping and intraspecies synteny analysis of all seven *ZmSPS* genes were performed using TBtools v2.376 software. The results showed that *ZmSPS* genes were unevenly distributed on six chromosomes (Figure 3), located on chr3, chr4, chr5, chr6, chr8, and chr9. Notably, chr4 contained two members (*ZmSPS2* and *ZmSPS3*), while the other chromosomes each carried a single member. This broad, cross-chromosomal distribution suggests that the *SPS* family has undergone considerable spatial diversification during maize evolution.

Intraspecies synteny analysis indicated that the expansion of the *ZmSPS* gene family primarily relied on segmental duplications across chromosomes. Three clear pairs of duplicated genes were identified and marked with colored lines in the Figure 3: *ZmSPS4*-*ZmSPS5* (chr5-chr6, blue), *ZmSPS1*-*ZmSPS6* (chr3-chr8, red), and *ZmSPS5*-*ZmSPS7* (chr6-chr9, purple). Interestingly, *ZmSPS5* was syntenic with both *ZmSPS4* and *ZmSPS7.* All three genes belong to Clade I in the phylogenetic tree, reflecting high evolutionary conservation. These results indicate that the expansion of the *ZmSPS* family is closely associated with whole-genome duplication (WGD) events in maize.

To investigate the evolutionary driving forces and potential functional divergence of the maize *ZmSPS* gene family, the nonsynonymous substitution rate (Ka), synonymous substitution rate (Ks), and their ratio (Ka/Ks) were calculated for the syntenic gene pairs. The results (Table S3) showed that all identified gene pairs had Ka/Ks < 1.0, ranging from 0.1133 to 0.2798. Among them, *ZmSPS4/7* exhibited the lowest value (0.1133), whereas *ZmSPS1/2* showed a relatively higher value (0.2798). These findings indicate that the *ZmSPS* gene family has undergone strong purifying selection during evolution, suggesting that family members tend to retain their original amino acid sequences to maintain functional stability as key enzymes in sucrose biosynthesis.

### 2.5. InterSpecies Synteny Analysis of the SPS Gene Family

**Figure 4:**
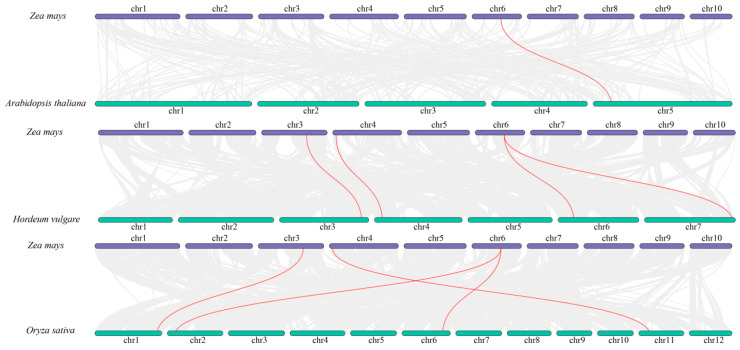
Synteny analysis of the *SPS* gene family in maize, Arabidopsis, barley, and rice. Red lines indicate syntenic gene pairs.

To explore the evolutionary conservation and interspecies homology of the maize *ZmSPS* gene family, synteny analysis was performed with Arabidopsis, rice, and barley (Figure 4). The results revealed that *ZmSPS* genes are highly conserved across both monocots and dicots, while more complex expansion patterns are observed within the Poaceae family. In comparison with Arabidopsis, only one significant orthologous pair was detected: *ZmSPS5* on maize chr6 exhibited synteny with *AT5G11110.1* on Arabidopsis chr5 (Table S4). In contrast, broader syntenic relationships were observed between maize and rice or barley. Four orthologous gene pairs were identified in rice, with genes on maize chr3, chr4, and chr6 corresponding to their respective rice chromosomes. Notably, the *ZmSPS* genes in poaceae displayed clear one-to-many expansion patterns. For example, *ZmSPS5* on maize chr6 corresponded to two transcripts in rice (*Os02t0184400-01* and *Os06t0643800-01*) and to chr6 and chr7 in barley, whereas genes on chr3 and chr4 corresponded to single orthologs in rice and barley. This distribution pattern reflects both expansion and conservation within the Poaceae. *ZmSPS5* on chr6 is the only member syntenic across all compared species, suggesting it represents a core ancestral gene of the *ZmSPS* family. In contrast, *SPS* members on chr3 and chr4 exhibit synteny only with rice and barley but are absent in Arabidopsis, indicating that these genes may have arisen through segmental duplication during the early evolution of monocots or poaceae and subsequently became stably inherited.

### 2.6. Phylogenetic Analysis of the Maize ZmSPS Gene Family

**Figure 5:**
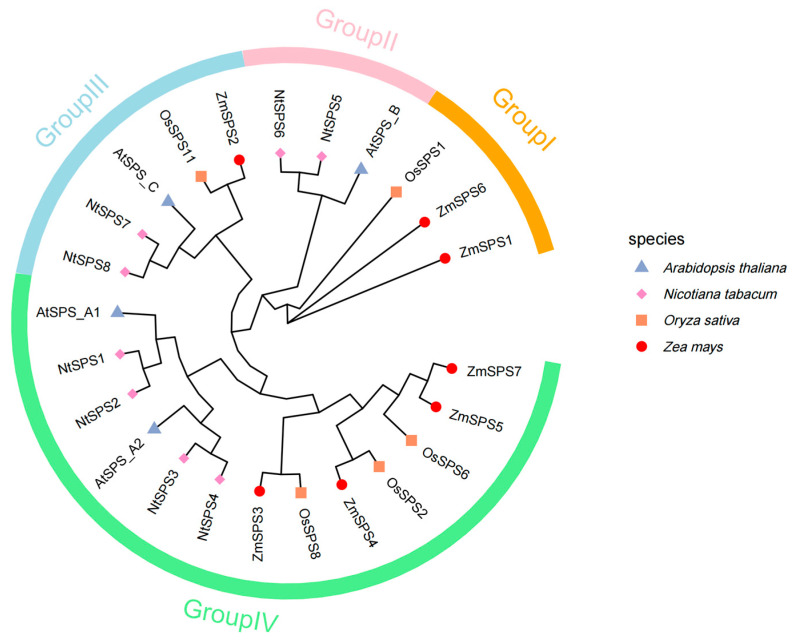
Phylogenetic tree of the *SPS* gene family. Triangles represent Arabidopsis, squares represent rice, circles represent maize, and diamonds represent tobacco. The outer ring of the tree is color-coded into four groups.

A phylogenetic tree was constructed based on the SPS protein sequences from maize, rice, Arabidopsis, and tobacco to clarify the evolutionary positions and relationships of *ZmSPS* family members (Figure 5). The analysis showed that SPS proteins from the four species were divided into four major groups (Group I–IV), exhibiting clear monocot–dicot differentiation patterns. Group I (yellow branch) included maize *ZmSPS1* and *ZmSPS6*, as well as rice *OsSPS1*, indicating high conservation within monocots. Group II (pink branch) contained *ZmSPS2*, which clustered with *AtSPS_B* from Arabidopsis and corresponding proteins from tobacco and rice, suggesting potential functional diversification. Group III (blue branch) mainly comprised members from Arabidopsis, tobacco, and rice, with no maize *ZmSPS* member detected, implying possible loss or functional degeneration during maize evolution. Group IV (green branch) was the largest subgroup, including maize *ZmSPS3*, *ZmSPS4*, *ZmSPS5*, and *ZmSPS7*. Maize members in this group formed close sister relationships with rice *OsSPS* members (*ZmSPS4* with *OsSPS8*, *ZmSPS5/7* with *OsSPS6*), reflecting high homology within the poaceae family.

Furthermore, the phylogenetic tree revealed clear species clustering: monocots (maize and rice) and dicots (Arabidopsis and tobacco) tended to cluster separately within each subgroup. In all branches containing maize members, *ZmSPS* preferentially clustered with rice *OsSPS*, consistent with the previously observed inter-species synteny results. This finding further confirms the close evolutionary relationship and high conservation of *SPS* genes between maize and rice.

### 2.7. miRNA-Mediated Regulatory Network Analysis of the Maize ZmSPS Gene Family

**Figure 6:**
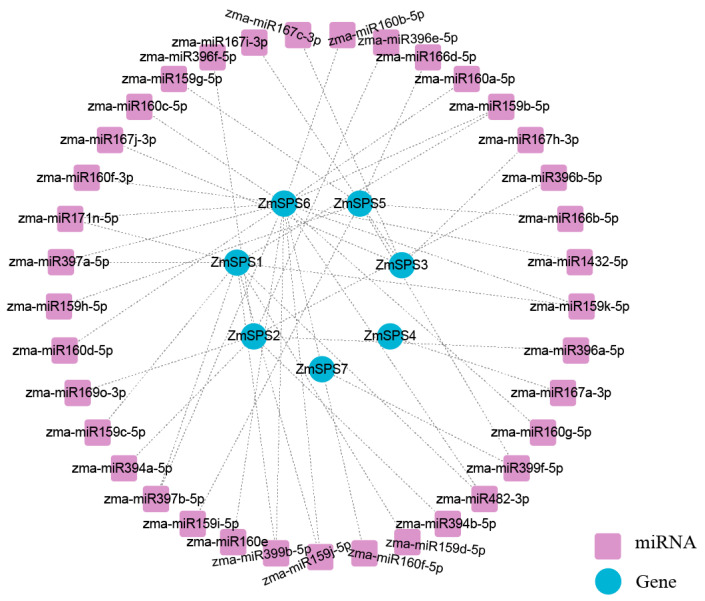
miRNA regulatory network of the maize *ZmSPS* gene family. Blue circles represent genes, purple squares represent miRNAs, and dashed lines indicate regulatory interactions.

To investigate the post-transcriptional regulation of the maize *ZmSPS* gene family, a miRNA-target regulatory network was predicted and constructed for the seven *ZmSPS* members. The results showed that this gene family is extensively regulated by multiple miRNAs (Figure 6), forming a highly interconnected many-to-many network, in which a single miRNA can target multiple *ZmSPS* genes, and each *ZmSPS* gene can be regulated by multiple distinct miRNAs. A total of 34 different miRNAs were identified, highlighting the complex post-transcriptional regulatory landscape of this family. Network analysis revealed differential regulation among the genes. *ZmSPS6*, *ZmSPS5*, and *ZmSPS1* were positioned at the network core, interacting with multiple miRNAs, such as members of the zma-miR167, zma-miR159, and zma-miR160 families. In addition, several specific regulatory interactions were observed, including *ZmSPS1* regulated by zma-miR397a-5p and ZmSPS3 regulated by zma-miR1432-5p, suggesting that these specific interactions may contribute to functional divergence in particular tissues or developmental stages.

### 2.8. GO Functional Enrichment Analysis of the Maize ZmSPS Gene Family

**Figure 7:**
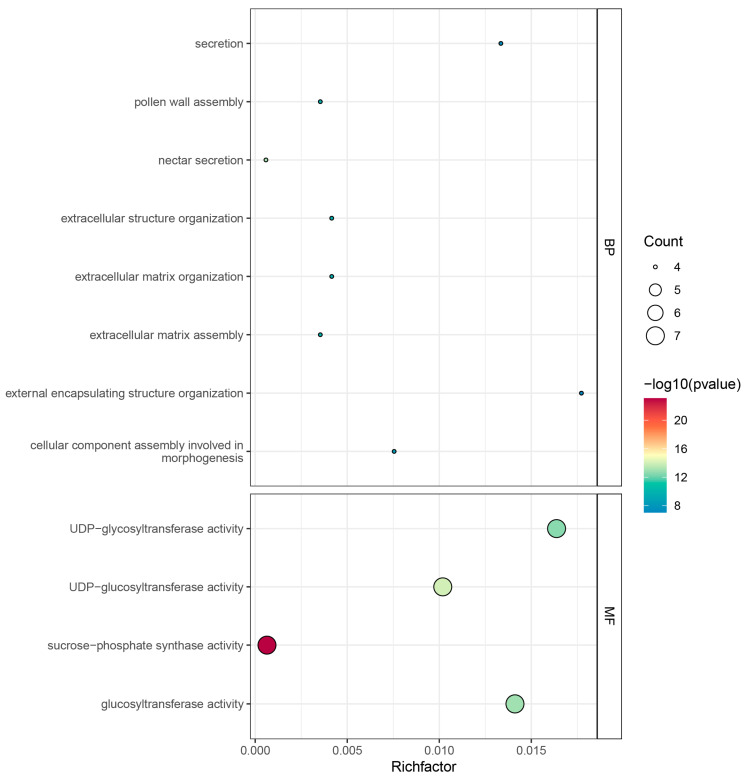
GO functional enrichment analysis of the *ZmSPS* gene family in maize. The *X*-axis represents the Richfactor, indicating the proportion of genes enriched in each pathway, with larger values reflecting higher enrichment. The *Y*-axis shows the names of the GO terms. The size of the dots represents the number of enriched genes, with larger dots indicating more genes enriched in the corresponding pathway. The color of the dots represents significance, where a higher −log10(*p*-value) corresponds to a smaller *p* value, indicating greater significance of the pathway.

To elucidate the biological functions of the *ZmSPS* family members in maize growth, development, and metabolic regulation, GO enrichment analysis was performed on the seven identified members (Figure 7). At the molecular function (MF) level, the family genes were primarily associated with catalytic activities, with the most significantly enriched term being sucrose-phosphate synthase activity. Additionally, *ZmSPS* genes were significantly enriched in UDP-glycosyltransferase activity and glucosyltransferase activity, reflecting their biochemical role in catalyzing sucrose-phosphate synthesis using UDP-glucose.

At the biological process (BP) level, *ZmSPS* genes were involved in multiple processes ranging from fundamental metabolism to complex developmental events. They were significantly enriched in processes such as secretion and nectar secretion, highlighting their central role in carbohydrate allocation and nectar formation. Furthermore, *ZmSPS* genes participated in extracellular structure organization, extracellular matrix assembly, and pollen wall assembly. Some members were enriched in cellular component assembly involved in morphogenesis, indicating that sucrose not only serves as an energy source but also acts as a precursor for the construction of cell wall-related structures.

### 2.9. Maize ZmSPS Gene Family Tissue Expression Pattern Analysis

**Figure 8:**
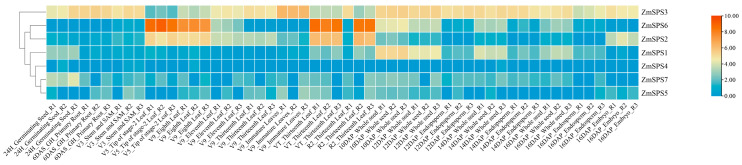
Heatmap showing tissue-specific expression patterns of *ZmSPS* genes. Samples include 24H_Germinating Seed (seeds at 24 h after germination), 6DAS_GH_Primary Root (primary roots at 6 days after germination), V3_Stem and SAM (stem and shoot apical meristem at the V3 stage), V5_Tip of Stage-2 Leaf (leaf tip of the second leaf at the V5 stage), V9_Immature Leaves (immature leaves at the V9 stage), V9_Eighth, V9_Eleventh, and V9_Thirteenth Leaves (fully expanded leaves at the V9 stage), VT_Thirteenth Leaf (thirteenth leaf at the tasseling stage), and R2_Thirteenth Leaf (thirteenth leaf at the R2 reproductive stage). Developing seed samples include whole seeds at 10, 12, 14, and 16 days after pollination (10 DAP, 12 DAP, 14 DAP, and 16 DAP), endosperm at 12, 14, and 16 DAP, and embryo at 16 DAP. Expression levels are shown as normalized values, and “–R1”, “–R2”, and “–R3” indicate three biological replicates for each sample.

The expression patterns of the seven *ZmSPS* family members were analyzed across different tissues and developmental stages using maize transcriptome data (Figure 8). The results revealed significant functional differentiation among family members, suggesting that they coordinately regulate maize metabolism through both division of labor and collaboration. *ZmSPS2* and *ZmSPS6* exhibited highly coordinated expression during leaf development. Both genes maintained very high transcript levels in leaf tips at the V5 stage, at the V9 stage, mature leaves at the VT stage, and leaves at the R2 stage, indicating that they are the core genes for sucrose synthesis in leaves, supporting plant growth and kernel filling. *ZmSPS3* displayed tissue-specific expression during immature tissue development, reaching its peak in V9 immature leaves. Similarly, *ZmSPS5* generally showed low expression across most tissues but had relatively higher expression in V9 immature leaves, suggesting a specific regulatory role during early leaf development. During seed germination and early developmental stages, *ZmSPS1* and *ZmSPS7* exhibited notable specificity. *ZmSPS1* showed the highest expression in 10 DAP whole seeds, primarily participating in sugar accumulation and allocation during early kernel development. *ZmSPS7* was most highly expressed in 24H germinating seeds, indicating its role in nutrient mobilization and energy supply during seed germination. *ZmSPS4* showed relatively low expression across most tissues.

Although all members belong to the same gene family, their expression varies markedly across tissues and developmental stages: *ZmSPS2* and *ZmSPS6* are highly expressed in leaves; *ZmSPS3* is relatively enriched in immature leaves, while *ZmSPS5* shows localized enrichment in young leaves; *ZmSPS1* and *ZmSPS7* are active during kernel development and germination; and *ZmSPS4* generally exhibits low expression, possibly being induced only under specific conditions.

### 2.10. ZmSPS Gene Family Transcriptome Expression Analysis Under Drought Stress

**Figure 9:**
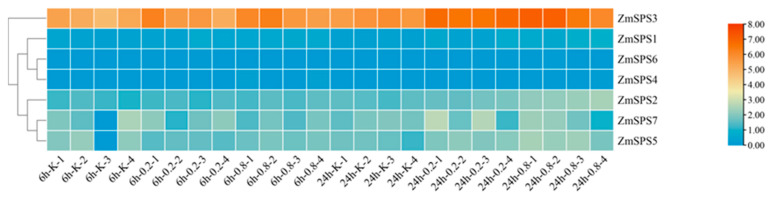
Mild and severe drought treatments were set at −0.2 MPa and −0.8 MPa, respectively, with distilled water treatment as the control. Seedlings were incubated in PEG solution or distilled water for 6 h and 24 h. Four biological replicates were included, yielding a total of 24 samples: for each time point (6 h and 24 h), there were 4 samples for the control, 4 samples for mild drought, and 4 samples for severe drought.

To evaluate the responsiveness of the maize SPS gene family under drought stress, transcriptome data from drought-treated plants were analyzed, examining the expression patterns of seven *ZmSPS* members under mild (−0.2 MPa) and severe (−0.8 MPa) PEG treatments at 6 h and 24 h. The results revealed significant differential expressions among family members in response to drought (Figure 9): *ZmSPS3* exhibited consistently high expression under drought stress. Its transcript levels were higher than those of other family members across all treatments and control, and expression increased significantly over time, reaching peak levels after 24 h under both mild and severe stress, indicating that *ZmSPS3* serves as a key regulatory gene in drought response. The branch members *ZmSPS2*, *ZmSPS5*, and *ZmSPS7* showed dynamic response patterns. *ZmSPS2* remained relatively stable throughout the treatments, with a slight upregulation observed at 24 h under severe stress. *ZmSPS5* and *ZmSPS7* displayed increasing trends under mild stress at both 6 h and 24 h, particularly pronounced at 24 h. The low-sensitivity members *ZmSPS1*, *ZmSPS4*, and *ZmSPS6* exhibited generally low expression under all treatments. These findings indicate considerable diversity among *ZmSPS* family members in spatial expression and in response to abiotic stress.

### 2.11. RT-qPCR Validation of the ZmSPS Gene Family Under PEG-Induced Osmotic Stress

**Figure 10:**
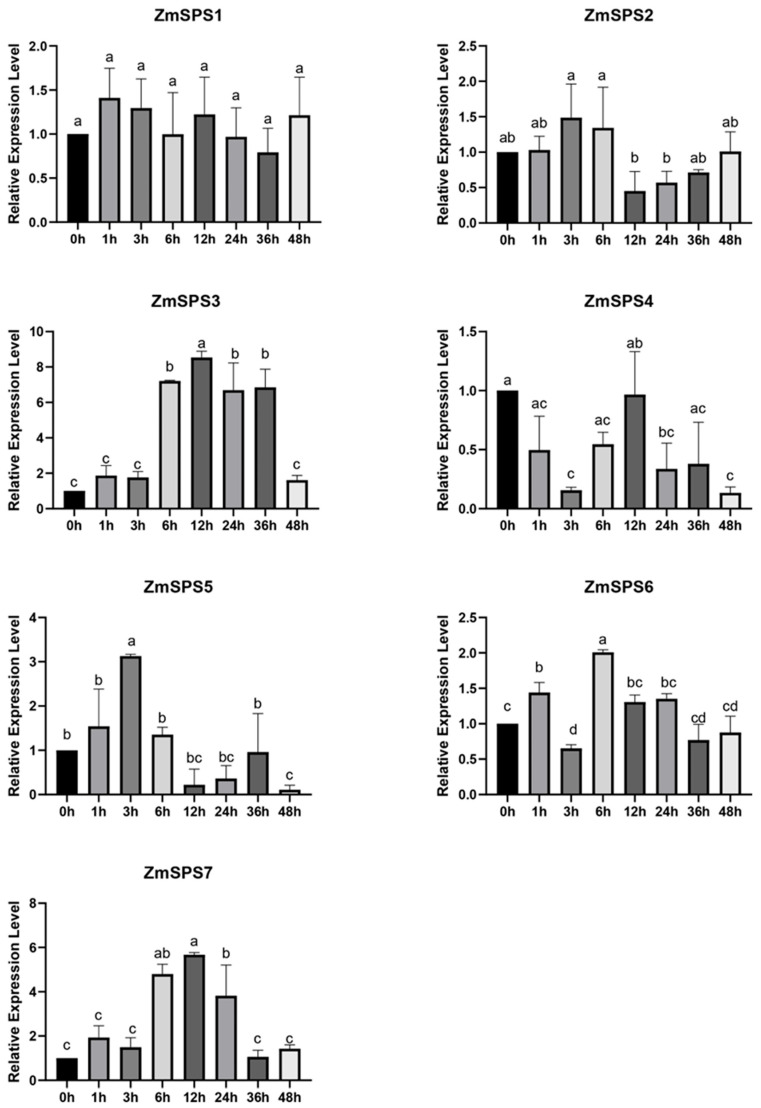
RT-qPCR analysis of the *ZmSPS* gene family in response to PEG-induced osmotic stress. The transcriptional levels of seven *ZmSPS* genes were measured at different time points (0, 1, 3, 6, 12, 24, 36, and 48 h) following simulated stress treatment. Expression levels were normalized to *ZmActin*. Bar graphs represent the means of three biological replicates, and error bars indicate standard deviation (SD). Different letters above bars indicate statistically significant differences at *p* < 0.05 according to one-way ANOVA; the same letters indicate no significant difference.

To verify the expression patterns of *ZmSPS* genes under osmotic stress conditions, the relative expression levels of the seven *ZmSPS* family members were analyzed by RT-qPCR during 0–48 h of PEG treatment. The results revealed distinct temporal expression patterns among the *ZmSPS* genes (Figure 10).

Among them, *ZmSPS3* exhibited the most pronounced response to stress treatment, with expression rapidly increasing at 6 h and peaking at 12 h (about 8.5-fold higher than the control), remaining relatively high at 24 h and 36 h. *ZmSPS7* displayed a similar trend, reaching its highest expression (about 6-fold) at 12 h before gradually declining. *ZmSPS5* showed early induction, peaking at 3 h (about 3-fold) and then gradually decreasing. *ZmSPS6* exhibited a transient increase (about 2-fold) at 6 h, then returned to lower levels. In contrast, *ZmSPS1* showed minimal change throughout the treatment period. *ZmSPS2* slightly increased at 3–6 h but decreased significantly after 12 h. *ZmSPS4* was initially suppressed under drought but showed partial recovery at 12 h. Overall, the RT-qPCR results were largely consistent with the transcriptome analysis, indicating that *ZmSPS* family members exhibit distinct dynamic expression patterns in response to osmotic stress.

### 2.12. ZmSPS3 Yeast Two-Hybrid (Y2H) Library Screening

Analysis based on transcriptome data showed that *ZmSPS3* exhibited strong induction expression characteristics under drought stress. Then, we performed RT-qPCR analysis on maize B73 seedlings treated with PEG-induced osmotic stress. The results showed that both *ZmSPS3* and *ZmSPS7* exhibited an upward expression trend; the response of *ZmSPS3* was the most significant, with its expression level at 12 h of treatment increasing by more than 8 times compared to 0 h, and its induction intensity ranked first among all family members. Considering that drought response is a complex process involving the synergistic action of multiple genes, this study integrated transcriptome and RT-qPCR and finally selected *ZmSPS3* as the target gene for subsequent research.

**Figure 11:**
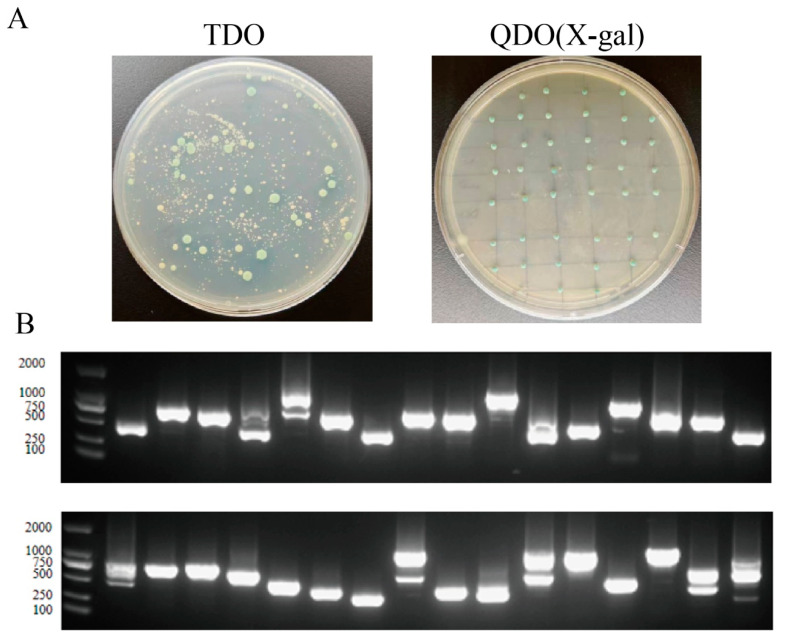
Yeast two-hybrid (Y2H) library screening of ZmSPS3. (**A**). Positive clones were initially screened on TDO plates, and randomly selected single clones were re-screened on QDO/X-gal plates. (**B**). PCR verification of single-clone colonies showed insert sizes all greater than 250 bp, with overall fragment lengths ranging from 500 to 1000 bp, consistent with the expected target fragment size distribution.

Self-activation testing of the *ZmSPS3* bait construct pGBKT7-*ZmSPS3* indicated that it does not possess transcriptional self-activation activity (Figure S1), making it suitable for subsequent Y2H screening. The pGBKT7-*ZmSPS3* bait was co-transformed with the maize Y2H library and screened on selective media. The DDO plates showed a sufficient number of single clones to meet the analysis requirements; positive clones from the primary screen were re-screened on TDO plates and grew normally on QDO/X plates while turning blue (Figure 11A), indicating reliable screening results.

PCR verification of the positive clones revealed insert sizes mainly ranging from 500 to 1000 bp, all exceeding 250 bp and meeting the library quality standards. Clones with clear bands were sequenced, yielding multiple valid sequences (Figure 11B). Through homology comparison and functional annotation, several candidate proteins potentially interacting with ZmSPS3 were identified (Table S5).

### 2.13. ZmSPS3 and Candidate Protein Yeast Two-Hybrid Validation

**Figure 12:**
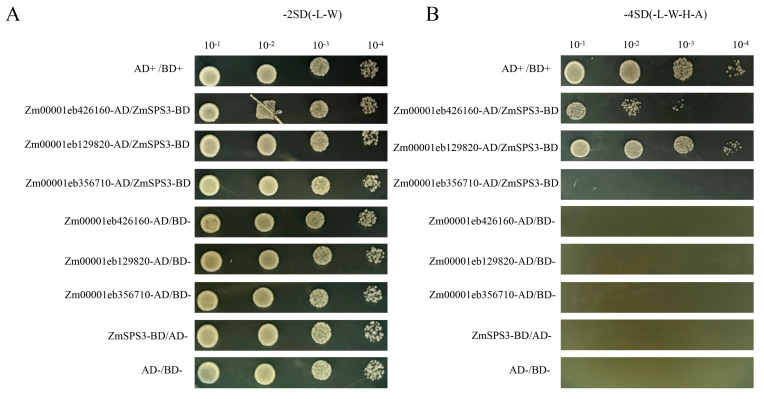
Verification of the interaction between ZmSPS3 and candidate proteins by yeast two-hybrid (Y2H) assay. (**A**). The AD vectors of candidate genes Zm00001eb426160, Zm00001eb129820, and Zm00001eb356710 were each co-transformed with the BD vector of ZmSPS3 into the yeast strain AH109. All co-transformants grew normally on the double-dropout medium SD/-Leu/-Trp (-2SD), indicating successful dual-plasmid transformation. (**B**). On the quadruple-dropout medium SD/-Leu/-Trp/-His/-Ade (-4SD), only the combinations Zm00001eb426160-AD/ZmSPS3-BD and Zm00001eb129820-AD/ZmSPS3-BD showed growth. The positive control (AD+/BD+) behaved as expected, while Zm00001eb356710-AD/ZmSPS3-BD and all negative controls did not grow, ruling out self-activation and non-specific interactions. Yeast cultures were adjusted to an initial OD600 of 1.0 and spotted in serial dilutions from 10^−1^ to 10^−4^.

To further verify the reliability of the Y2H library screening results, a pairwise yeast two-hybrid assay was performed to examine the interactions between ZmSPS3 and the candidate proteins: a protein kinase (Zm00001eb129820), an F-box protein (Zm00001eb426160), and RABD2C (Zm00001eb356710). The bait vector ZmSPS3-BD was co-transformed with the AD vectors of the candidate proteins (Zm00001eb129820-AD, Zm00001eb426160-AD, and Zm00001eb356710-AD) into yeast cells (Figure 12). All co-transformants grew normally on DDO. On QDO, these co-transformants continued to grow robustly and formed clear colonies, indicating stable interactions between ZmSPS3 and the two candidate proteins. In contrast, all negative controls failed to grow on QDO medium, effectively ruling out autoactivation or nonspecific interactions. These results demonstrate that ZmSPS3 can specifically interact with the protein kinase and F-box protein in yeast cells.

## 3. Discussion

The number of *SPS* gene family members in maize has previously been reported as six or seven [20,29]. In this study, a total of seven members were systematically identified, which are mainly distributed across six chromosomes, and encode proteins of approximately 120 kDa. The Ka/Ks ratios for all gene pairs are well below 1, indicating that this gene family has undergone strong purifying selection during evolution. Phylogenetic analysis and cross-species synteny analysis revealed that maize *SPS* genes are closely related to those in other monocotyledons, such as rice and barley, whereas they are more distantly related to dicotyledons like Arabidopsis and tobacco, suggesting that maize SPS genes may have functions highly similar to those in rice and barley.

Sucrose metabolism is a central process in plant responses to abiotic stress [10,18]. In this study, although both *ZmSPS3* and *ZmSPS7* were induced by stress, the response of *ZmSPS3* was the most prominent. Notably, the expression of ZmSPS3 increased by more than 8-fold at 12 h compared to 0 h under PEG treatment. Notably, *ZmSPS3* showed a strong correlation between the enrichment of stress responsive *cis*-elements in its promoter region and its transcriptional response, suggesting that it may serve as a key rate-limiting gene in regulating carbon allocation and the accumulation of osmoprotectants in maize under drought conditions.

Analysis of *cis*-acting elements in the promoter regions of the *ZmSPS* gene family revealed that these genes not only contain drought-responsive MBS elements but are also enriched with various hormone-responsive motifs, such as the abscisic acid (ABA) responsive element ABRE, the methyl jasmonate (MeJA) responsive element TGACG-motif, and the auxin-responsive element AuxRR-core. The presence of ABRE elements in *ZmSPS* promoters provides a molecular basis for the coordination between sucrose and ABA signaling. For example, in grape, sucrose can synergistically induce the expression of the maturation-related gene *ASR* together with ABA [30,31]. ABA signaling not only directly activates *ZmSPS* transcription, but the resulting sucrose may further act as a secondary messenger to feedback and enhance the ABA signaling pathway [30]. As shown in fruit ripening studies, sucrose can increase ABA levels by inducing ABA biosynthesis genes (*VvNCED2*) and repressing ABA catabolic genes (*VvCYP707A*) [32]. Goren et al. found that suppressing sucrose synthase genes in tomato had little effect on total sucrose and other soluble sugars but increased the expression of the auxin transport-related gene *PIN1* in shoot apices and leaves [33]. These findings suggest that the *ZmSPS* family may function in multiple signaling pathways, participating not only in sucrose metabolism but also in modulating plant growth, development, and stress responses through interactions with ABA, auxin, and other hormone signaling pathways.

In this study, yeast two-hybrid assays verified that ZmSPS3 physically interacts with a protein kinase and an F-box protein. This finding extends research on maize SPS from solely transcriptional regulation to the complex layer of post-translational regulation. Previous studies have shown that in rice, OsCPK17 can phosphorylate OsSPS4, participating in low-temperature stress responses [34]. In addition, multiple phosphorylation sites have been reported in SPS proteins, such as Ser^158^ [35], Ser^229^ [36], and Ser^424^ [37]. Based on the yeast two-hybrid library results for ZmSPS3 in this study, its interaction with the protein kinase suggests the possibility of phosphorylation modifications. In further studies, we will employ phosphorylation site prediction and protein experiments to validate the interaction between the protein kinase and ZmSPS3 and to elucidate its role in drought stress response.

F-box proteins are core components of the SCF E3 ubiquitin ligase complex. The interaction between ZmSPS3 and the F-box protein suggests that plants may finely regulate ZmSPS3 degradation via the ubiquitin-proteasome pathway. Moreover, sucrose can regulate the ethylene signaling negative regulator CONSTITUTIVE TRIPLE RESPONSE1 (CTR1) through the F-box protein ZEITLUPE, thereby stabilizing GIGANTEA (GI) and maintaining a normal circadian rhythm in plants [38]. This mechanism allows plants to rapidly reduce SPS protein abundance when drought is alleviated or environmental conditions change, thus preventing feedback inhibition or metabolic imbalance caused by excessive sucrose accumulation.

However, several limitations in the present study should be acknowledged. First, the osmotic stress simulated by PEG treatment, while informative, does not fully replicate the physiological complexity of soil-based drought stress in field conditions. Second, our functional insights into *ZmSPS3* are currently limited to transcriptomic responses and protein–protein interactions, lacking direct evidence from physiological and biochemical measurements, such as SPS enzymatic activity and sucrose content, as well as photosynthetic phenotypic data. Consequently, further investigations involving stable genetic transformation and comprehensive phenotypic characterization under more realistic drought conditions are required. Future research will focus on evaluating the growth performance and water-use efficiency of transgenic maize lines to fully elucidate the biological role of *ZmSPS3* in the complex drought-response regulatory networks.

## 4. Materials and Methods

### 4.1. Plant Material and Drought Stress Treatment

The maize inbred line B73 was used as the experimental material. Seeds were soaked in distilled water for 24 h and then placed in a dark incubator at 37 °C for germination. After germination, seedlings were transplanted into seedling pots containing a 1:1 (*v*/*v*) mixture of perlite and soil. Plants were grown in a controlled-environment growth chamber with day/night temperatures of 25 °C/22 °C and a 16 h/8 h light/dark photoperiod. When seedlings reached the three-leaf stage, osmotic stress was simulated using a 12% PEG6000 solution to mimic drought conditions (Coolaber, Beijing, China) [39]. Leaf samples were collected at 0, 1, 3, 6, 12, 24, 36, and 48 h after treatment. Each time point included three biological replicates, with each replicate weighing approximately 0.1 g, and three technical replicates were set for each biological sample. All collected leaf samples were immediately frozen in liquid nitrogen and then stored at −80 °C for subsequent analyses.

### 4.2. Identification of the ZmSPS Gene Family

The maize genome (Zm-B73-REFERENCE-NAM-5.0), protein sequences, and annotation files were downloaded from the Phytozome database (https://phytozome-next.jgi.doe.gov/, accessed on 4 October 2025). Members of the *SPS* gene family were initially identified using HMMER v3.3 based on Hidden Markov Models (HMMs) for the *SPS* family (PF00534, PF00862, PF05116) by searching against the maize protein sequences [40]. In addition, BLASTP version 2.15.0 searches were performed using *Oryza sativa* (rice) SPS protein sequences against the maize protein dataset, and the results were combined to generate a list of candidate gene IDs. The candidate sequences were further confirmed for the presence of conserved SPS domains using SMART (http://smart.embl.de/, accessed on 4 October 2025), the NCBI Conserved Domain Database (https://www.ncbi.nlm.nih.gov/cdd/, accessed on 4 October 2025), Pfam (http://pfam.xfam.org/, accessed on 4 October 2025), and Phmmer (https://www.ebi.ac.uk/Tools/hmmer/search/phmmer, accessed on 4 October 2025). Sequences lacking core *SPS* domains were discarded, and the remaining sequences were designated as the final set of *ZmSPS* genes.

### 4.3. Physicochemical Properties and Subcellular Localization Prediction of the ZmSPS Gene Family

The physicochemical properties of ZmSPS proteins were analyzed using the ProtParam online tool (https://web.expasy.org/protparam/, accessed on 5 October 2025). Subcellular localization of SPS proteins was predicted using DeepLoc-2.0 (https://services.healthtech.dtu.dk/services/DeepLoc-2.0/, accessed on 5 October 2025).

### 4.4. Conserved Domain and Gene Structure Analysis of ZmSPS Gene Family

The conservation of ZmSPS protein sequences was analyzed using the MEME Suite 5.59 online tool (https://meme-suite.org/meme/tools/meme, accessed on 5 October 2025). The number of motifs to be identified was set to 15, with motif widths constrained between 6 and 100 residues. Based on the maize genome GFF annotation file, exon and intron distribution information of the ZmSPS genes was extracted. Gene structures were subsequently visualized using appropriate visualization tools.

### 4.5. Prediction of Cis-Elements in the Promoters of the ZmSPS Gene Family

Based on the maize whole-genome sequence and corresponding annotation files, the 2000 bp upstream sequences from the start codon (ATG) of each *ZmSPS* gene were extracted as the putative promoter regions. These sequences were submitted to the PlantCARE online database (https://bioinformatics.psb.ugent.be/webtools/plantcare/html/, accessed on 10 October 2025) for identification and functional annotation of *cis*-acting elements. The distribution of core *cis*-elements was integrated and visualized using the R programming language (version 4.3.1). Additionally, potential miRNA targets of *ZmSPS* genes were predicted using the psRNATarget online tool (https://www.zhaolab.org/psRNATarget/, accessed on 10 October 2025) with default parameters.

### 4.6. Chromosomal Localization and Evolutionary Synteny Analysis

Based on genome annotation information, the physical distribution of *ZmSPS* genes on the 10 maize chromosomes was visualized using TBtools v2.376 [41]. Multiple sequence alignment of SPS protein sequences from maize, rice, Arabidopsis, and tobacco (*Nicotiana tabacum*) was performed using MAFFT v7.526 [42]. Phylogenetic trees were constructed using IQ-TREE (v3) based on the Maximum Likelihood (ML) method [43]. The best-fit substitution model was automatically selected using ModelFinder. To assess the reliability of tree branches, ultrafast bootstrap (-bb) analysis was performed with 1000 replicates. Phylogenetic trees were visualized and beautified using Evolview3 (https://www.evolgenius.info/evolview-v3/, accessed on 10 October 2025). Synteny analysis was conducted using TBtools to assess both intra-genome collinearity within maize and inter-species collinearity with rice (*Oryza sativa*), barley (*Hordeum vulgare*), and Arabidopsis (*Arabidopsis thaliana*) genomes, which were downloaded for this purpose.

### 4.7. Transcriptome Analysis of the ZmSPS Gene Family

Transcriptome datasets with accession numbers SRP010680 and PRJNA226757 were retrieved from the NCBI database (https://www.ncbi.nlm.nih.gov/, accessed on 10 October 2025). Gene expression of *ZmSPS* members at different developmental stages and under drought stress was quantified using TPM (Transcripts Per Million) values after alignment with Salmon V1.10.3 software [44]. Visualization and hierarchical clustering of gene expression patterns were performed using the heatmap module of TBtools.

### 4.8. Quantitative Real-Time PCR (RT-qPCR) and Data Analysis

To further validate the expression patterns of *ZmSPS* genes, total RNA was extracted from maize leaves using FreeZol Reagent (Vazyme, Nanjing, China). The RNA was then reverse transcribed into cDNA using HiScript III RT SuperMix for qPCR (+gDNA wiper) (Vazyme, Nanjing, China). Primers for RT-qPCR were designed using NCBI Primer-BLAST (https://www.ncbi.nlm.nih.gov/tools/primer-blast/, accessed on 20 October 2025), and their sequences are listed in Table S1. RT-qPCR reactions were performed using ChamQ Universal SYBR qPCR Master Mix (Vazyme, Nanjing, China) on a QuantStudio 3 real-time PCR system, following the protocol described by Mo et al. [45]. Relative gene expression levels were calculated using the LV method [46] and normalized against *ZmActin* as the internal reference gene (Table S1) [47]. Each sample included three biological replicates and three technical replicates. Statistical analyses were conducted using SPSS software (v26.0), and graphs were generated with GraphPad Prism 9.0.

### 4.9. GO Annotation and Functional Enrichment Analysis of ZmSPS Genes Family

The maize whole-genome protein sequences were functionally annotated using the EggNOG-mapper v5 online platform (http://eggnog5.embl.de/#/app/home, accessed on 25 October 2025) to obtain detailed Gene Ontology (GO) annotations for *ZmSPS* family members. Based on the annotation results, functional enrichment analysis was performed using the clusterProfiler R package (v4.0) [48]. The analysis covered three core GO categories: biological process (BP), cellular component (CC), and molecular function (MF). GO terms related to stress responses and resistance were further visualized using the ggplot2 (version 3.5.1) R (version 4.3.1) package, providing an intuitive representation of the biological functions of the *ZmSPS* gene family.

### 4.10. Yeast Two-Hybrid Screening of ZmSPS3

The full-length coding sequence (CDS) of *ZmSPS3* was cloned into the pGBKT7 vector to construct the bait plasmid pGBKT7-*ZmSPS3* (Table S1). Using the PEG/LiAc method, the bait plasmid was co-transformed with the empty pGADT7 vector into Y2HGold yeast competent cells [49]. Transformed yeast strains were plated on double dropout (SD/-Leu-Trp, DDO) and quadruple dropout (SD/-Leu-Trp-His-Ade, QDO) media to evaluate the transcriptional autoactivation activity and potential cytotoxicity of the bait protein. The pGBKT7-*ZmSPS3* bait strain was subsequently co-transformed with a maize whole-tissue cDNA library. Transformants were first plated on TDO medium for primary screening, and colony-forming units (CFUs) were used to assess library coverage. Positive clones from the primary screen were transferred to QDO plates containing X-Gal for stringent secondary screening. Clones showing healthy growth and blue coloration were selected as potential interacting candidates. PCR amplification of positive clones from the secondary screen was performed using vector-specific primers Y2H-F/Y2H-R (Table S1). PCR products with clear and single bands were recovered and sequenced. The resulting sequences were analyzed for homology and functional annotation using NCBI BLAST and the UniProt database. Additionally, sequencing results were compared against the MaizeGDB database (https://maizegdb.org/, accessed on 30 October 2025) to identify candidate proteins potentially interacting with ZmSPS3. The list of aligned genes and functional annotation results are presented in the corresponding Table S5.

### 4.11. Yeast Two-Hybrid Validation

To validate the physical interactions between ZmSPS3 and candidate proteins, the full-length CDS of *Zm00001eb426160*, *Zm00001eb129820*, and *Zm00001eb356710* were individually cloned into the pGADT7 vector (Table S1). The resulting prey plasmids were co-transformed with the pGBKT7-*ZmSPS3* bait plasmid into Y2H yeast competent cells. Transformants were plated on double dropout (SD/-Leu-Trp, DDO) medium and quadruple dropout (SD/-Leu-Trp-His-Ade, QDO) stringent selection medium. Direct interactions between ZmSPS3 and each candidate protein were confirmed based on colony growth on QDO medium.

## 5. Conclusions

In this study, the *SPS* gene family in maize was systematically identified using bioinformatics approaches, resulting in the characterization of seven *ZmSPS* members. Their gene structures, conserved motifs, promoter elements, evolutionary relationships, and synteny were comprehensively analyzed. GO annotation and miRNA target prediction further revealed the potential transcriptional regulatory network of the *SPS* genes. Combined with transcriptome analysis under PEG-induced osmotic stress and validation by RT-qPCR, the results showed that *ZmSPS3* was significantly upregulated under drought, with expression levels continuously increasing over time, indicating its key role in maize drought response. Furthermore, yeast two-hybrid experiments suggested interactions of ZmSPS3 with proteins, including a protein kinase and an F-box protein, pointing toward possible regulatory mechanisms involving post-translational modification and protein stability. Overall, these findings enrich the functional annotation of the maize *SPS* gene family and provide preliminary experimental evidence for constructing its molecular regulatory network under drought stress conditions.

## Figures and Tables

**Table 1:** Physicochemical properties of the maize *ZmSPS* gene family.

Gene_id	Name	Protein Length	CDS Length (nt)	Molecular Weight (Da)	Isoelectric Point (pI)	Hydrophobicity (GRAVY)	Subcellular Localization
Zm00001eb143360	*ZmSPS1*	1079	3237	119417.73	6.22	−0.4	Cytoplasm
Zm00001eb167910	*ZmSPS2*	1051	3153	114276.78	6.65	−0.25	Cytoplasm
Zm00001eb177450	*ZmSPS3*	1059	3177	118243.38	6.04	−0.42	Cytoplasm
Zm00001eb234400	*ZmSPS4*	951	2853	106600.36	7.38	−0.33	Cytoplasm
Zm00001eb275240	*ZmSPS5*	975	2925	109210.39	7.03	−0.3	Cytoplasm
Zm00001eb364620	*ZmSPS6*	1068	3204	118573.84	6.22	−0.39	Cytoplasm
Zm00001eb387040	*ZmSPS7*	844	2532	94499.1	6.75	−0.2	Cytoplasm

## Data Availability

The transcriptome datasets analyzed in this study are publicly available in the NCBI Sequence Read Archive (SRA) and BioProject databases under accession numbers SRP010680 and PRJNA226757.

## Supplementary Materials

The following supporting information can be downloaded at: https://www.mdpi.com/article/10.3390/plants15060885/s1. Table S1. Primers used for ZmSPS gene amplification, Table S2. ZmSPS exon and intron number, Table S3. ZmSPS Ka/Ks, Table S4. Synteny analysis of the ZmSPS gene family based on MCScanX, Table S5. Functional annotation of genes identified from yeast library screening of ZmSPS3, Figure S1. Verification of auto-activation of the tested constructs in the yeast two-hybrid system.
