# Supplementary material for: Identification and Drought-Responsive Expression Analysis of the ZmSPS Gene Family in Maize and Preliminary Investigation of the ZmSPS3 Regulatory Network
Source: Plants (Basel). 2026 Mar 12;15(6):885. doi: 10.3390/plants15060885 (PMC13029493; doi:10.3390/plants15060885)
Supplement: Supplementary file 1 [file plants-15-00885-s001.zip › plants-4178353-supplementary.pdf]

Table S1. Primers used for *ZmSPS* gene amplification

| Primer name | Sequence (5'–3') |
|---|---|
| *ZmSPS1-F* | GGTTAAGGAACCCGAGGTGG |
| *ZmSPS1-R* | GACGACAATGGGTCGTTGGT |
| *ZmSPS2-F* | CCCTAAGCACCACAAGCAGA |
| *ZmSPS2-R* | TCTATGAGGGTGAGGCCGAA |
| *ZmSPS3-F* | CGGGAACGACTGGATCAACA |
| *ZmSPS3-R* | CCTCCTCGACGAAGTATCGC |
| *ZmSPS4-F* | AGTGGCATACCCCAAGCATC |
| *ZmSPS4-R* | CCGCAATGGCATTCTGATTAATTT |
| *ZmSPS5-F* | CGACCTCTACAAGACCTGGC |
| *ZmSPS5-R* | CTCTAGACGGCGTTTGGACA |
| *ZmSPS6-F* | CATGGACGCTGAAGGAAAGC |
| *ZmSPS6-R* | TCTTTGATGAGGAACGCGACA |
| *ZmSPS7-F* | CCTACGGTGACAGCACAACT |
| *ZmSPS7-R* | TCTCTCCACGTATCAGGCCA |
| *ZmActin1-F* | TACGAGATGCCTGATGGTCAGGTCA |
| *ZmActin1-R* | TGGAGTTGTACGTGGCCTCATGGAC |
| *ZmSPS3-BD-EcoRI-F* | CATGGAGGCCGAATTCATGGCCGGGAACGACT |
| *ZmSPS3-BD-BamHI-R* | GCAGGTCGACGGATCCGTCTTTCAATATACCAAATT GCCGTAGAG |
| *Zm00001eb129820-AD-EcoRI-F* | GGAGGCCAGTGAATTCATGGTGCTTGAGTTTGTCAA CGG |
| *Zm00001eb129820-AD-BamHI-R* | CGAGCTCGATGGATCCGAACCCAGATAGAGGGAAC GC |
| *Zm00001eb356710-AD-EcoRI-F* | GGAGGCCAGTGAATTCATGAATCCCGAGTACGACTA CCT |
| *Zm00001eb356710-AD-BamHI-R* | CGAGCTCGATGGATCCAGAGCAGCAGCTGCTTTG |
| *Zm00001eb426160-AD-EcoRI-F* | GGAGGCCAGTGAATTCATGGGCCACTCCCCCT |
| *Zm00001eb426160-AD-BamHI-R* | CGAGCTCGATGGATCCTGGTGGCAGAGGATTGCCA |
| *Y2H-F* | ATGGAGTACCCATACGACGTAC |
| *Y2H-R* | TTGAGATGGTGCACGATGC |

Table S2. ZmSPS exon and intron number

| gene | Exon_number | Intron_number |
|---|---|---|
| *ZmSPS1* | 12 | 11 |
| *ZmSPS2* | 12 | 11 |
| *ZmSPS3* | 14 | 13 |
| *ZmSPS4* | 13 | 12 |
| *ZmSPS5* | 14 | 13 |
| *ZmSPS6* | 12 | 11 |
| *ZmSPS7* | 13 | 12 |

Table S3.ZmSPS Ka/Ks

| Seq_1 | Seq_2 | Ka | Ks | Ka_Ks |
|---|---|---|---|---|
| ZmSPS1 | ZmSPS6 | 0.022307252028157386 | 0.19301713365525938 | 0.11557135683094086 |
| ZmSPS1 | ZmSPS2 | 0.3855549751507449 | 1.377887836605377 | 0.2798159363251326 |
| ZmSPS2 | ZmSPS6 | 0.37476303983622294 | 1.4126720970925195 | 0.2652866440892679 |
| ZmSPS3 | ZmSPS5 | 0.2511442822858324 | 1.31704874932356 | 0.19068715749118686 |
| ZmSPS3 | ZmSPS4 | 0.23962980055680025 | 1.5559342442677049 | 0.15401023625492682 |
| ZmSPS3 | ZmSPS7 | 0.2610182289778366 | 1.4540227522700244 | 0.17951454237585637 |
| ZmSPS4 | ZmSPS5 | 0.0964930612925454 | 0.8385810242736045 | 0.11506706984710226 |
| ZmSPS4 | ZmSPS7 | 0.10068342239982052 | 0.8885365285001565 | 0.11331376839371302 |

Table S4. Synteny analysis of the ZmSPS gene family based on MCScanX

| Species | Chromosome | Gene ID | Corresponding species | Chromosome | Orthologous gene ID |
|---|---|---|---|---|---|
| *Zea mays* | chr3 | *ZmSPS1* | *Oryza sativa* | chr1 | *Os01t0919400-01* |
| | chr4 | *ZmSPS2* | | chr11 | *Os11t0236100-01* |
| | chr6 | *ZmSPS5* | | chr2 | *Os02t0184400-01* |
| | chr6 | *ZmSPS5* | | chr6 | *Os06t0643800-01* |
| *Zea mays* | chr6 | *ZmSPS5* | *Arabidopsis thaliana* | chr5 | *AT5G11110.1* |
| *Zea mays* | chr3 | *ZmSPS1* | *Hordeum vulgare* | chr3 | *HORVU.MOREX.r3.3HG0309930.1* |
| | chr4 | *ZmSPS2* | | chr4 | *HORVU.MOREX.r3.4HG0344630.1* |
| | chr6 | *ZmSPS5* | | chr6 | *HORVU.MOREX.r3.6HG0568070.1* |
| | chr6 | *ZmSPS5* | | chr7 | *HORVU.MOREX.r3.7HG0745120.1* |

Table S5. Functional annotation of genes identified from yeast library screening of ZmSPS3

| transcriptName | Pfam | GO | Annotation |
|---|---|---|---|
| Zm00001eb356710_T001 | PF00025 | GO:0003924; GO:0005525 | Ras-related protein RABD2C |
| Zm00001eb035570_T001 | | | |
| Zm00001eb299760_T001 | PF01789; PF20928 | GO:0005509; GO:0009523; GO:0009654; GO:0015979; GO:0019898 | Oxygen-evolving enhancer protein 2-1 |
| Zm00001eb202760_T001 | PF00227; PF10584 | GO:0005839; GO:0006511; GO:0019773; GO:0051603 | 20S proteasome subunit alpha 4 |
| Zm00001eb407630_T001 | PF01419 | | Jacalin-related lectin 19 |
| Zm00001eb319820_T001 | PF00388 | GO:0006629; GO:0008081 | Phosphatidylinositol phospholipase C |
| Zm00001eb145440_T001 | PF16544 | GO:0003676; GO:0003723 | GH05812P-related protein |
| Zm00001eb336580_T001 | PF02605 | GO:0009522; GO:0009538; GO:0015979 | Photosystem I subunit XI (psaL) |
| Zm00001eb168670_T001 | PF00454; PF21245 | | Phosphatidylinositol 4-kinase |
| Zm00001eb363610_T001 | | | Heparan-alpha-glucosaminide N-acetyltransferase |
| Zm00001eb022980_T001 | PF01619 | | Proline dehydrogenase |
| Zm00001eb106460_T001 | PF00125; PF16211 | GO:0000786; GO:0003677; GO:0030527; GO:0046982 | Histone H2A |
| Zm00001eb230760_T001 | | | Protein LPA2 |
| Zm00001eb143640_T001 | PF04398 | | |
| Zm00001eb168890_T001 | | | |
| Zm00001eb306790_T001 | PF01255 | GO:0016765 | Dehydrodolichyl diphosphate synthase |
| Zm00001eb129820_T001 | PF00069; PF03822 | GO:0004672; GO:0004713; GO:0005524; GO:0006468; GO:0007165 | Protein kinase (Pkinase / NAF domain) |
| Zm00001eb268360_T001 | PF13473 | GO:0005507; GO:0009055 | Plastocyanin (petE) |
| Zm00001eb061730_T001 | | | |
| Zm00001eb212520_T001 | PF02531 | GO:0009522; GO:0009538; GO:0015979 | Photosystem I subunit II (psaD) |
| Zm00001eb233170_T001 | PF00504 | | Light-harvesting complex I chlorophyll a/b binding protein 1 |
| Zm00001eb426160_T001 | PF12937 | GO:0005515 | F-box protein SKIP2 |
| Zm00001eb426150_T001 | PF18511 | GO:0005515 | F-box protein SKIP2 |
| Zm00001eb421460_T001 | PF00355; PF02921 | GO:0008121; GO:0016020; GO:0051537 | Quinol--cytochrome c reductase |
| Zm00001eb098610_T001 | | | Expressed protein |

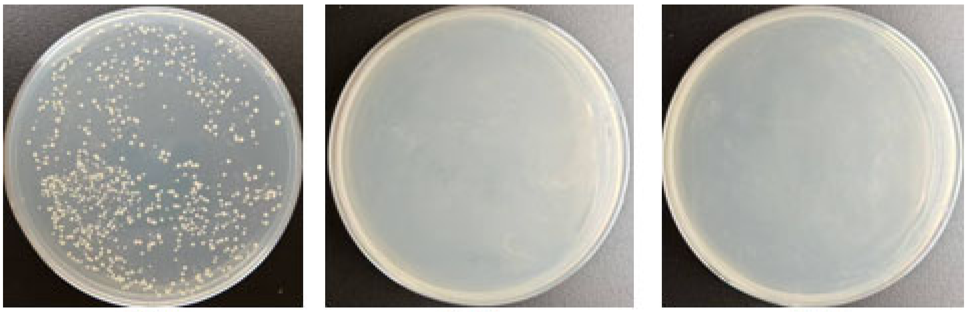

DDO TDO QDO

Figure S1. Verification of auto-activation of the tested constructs in the yeast two-hybrid system.
